# A novel liposomal drug delivery system for PMMA bone cements

**DOI:** 10.1002/jbm.b.33488

**Published:** 2015-08-10

**Authors:** Wayne Nishio Ayre, James C. Birchall, Samuel L. Evans, Stephen P. Denyer

**Affiliations:** ^1^ School of DentistryCardiff UniversityCardiffCF14 4XYUK; ^2^ School of Pharmacy and Pharmaceutical SciencesCardiff UniversityCardiffCF10 3NBUK; ^3^ School of EngineeringCardiff UniversityCardiffCF24 3AAUK; ^4^ School of Pharmacy and Biomolecular SciencesBrightonBN2 4GJUK

**Keywords:** bone cement, PMMA, acrylic, antimicrobial, controlled release, infection, joint replacement

## Abstract

The population in developed countries is ageing and the number of people experiencing joint‐related conditions, such as osteoarthritis, is expected to increase. Joint replacements are currently the most effective treatment for severe joint conditions and although many of these procedures are successful, infection developing after the procedure is still an issue, requiring complex and expensive revisions. Whilst incorporating a powdered antibiotic within the bone cement can reduce infection rates, the powder frequently agglomerates, resulting in poor antibiotic release characteristics and compromised mechanical performance of the cement. To overcome these issues, a novel delivery system consisting of antibiotic‐loaded nano‐sized liposomes was developed for inclusion into polymethyl methacrylate (PMMA) bone cement. This system was tested in a commercial cement (Palacos R) and consistently delivered a higher percentage (22%) of the incorporated antibiotic when compared to the powdered antibiotic cement (9%), meaning less antibiotic needs to be incorporated than with conventional cement. The novel system resulted in a controlled and gradual release of antibiotic over a longer, 30‐day period and enhanced the toughness, bending strength and Vickers hardness of the cement, without altering its polymerization or molecular structure. This new material has the potential to significantly reduce infections in cemented joint replacements leading to enhanced patient quality of life and reduced healthcare costs. © 2015 Wiley Periodicals, Inc. J Biomed Mater Res Part B: Appl Biomater, 104B: 1510–1524, 2016.

## INTRODUCTION

Polymethyl methacrylate (PMMA) bone cements have been widely employed in hip and knee replacements since their introduction by Sir John Charnley in 1953.^1^, ^2^ In England and Wales ∼178,000 hip and knee replacements, of which 102,700 were cemented, were performed in 2012, a 300% increase since 2004.^3^ Bone cement has several roles: to secure the implant in place; to distribute stresses between the stiff metallic implant and the bone; and, frequently, to prevent infection by releasing an antibiotic. The latter role was first developed in 1969 when Buchholz et al.^4^ observed that the monomer of bone cement was eluted over long periods of time after setting. This led to the concept of local delivery of powdered antimicrobial agents from bone cement. The most successful of the antimicrobial agents tested is gentamicin sulfate, a broad spectrum antibiotic, which is currently employed in several commercial bone cements. Gentamicin is an aminoglycoside antibiotic that inhibits protein synthesis by binding to the 30S ribosome subunit of bacteria.^5^ Gentamicin is available as a dry powder and is heat stable, making it ideal for the high setting temperatures of bone cement.^5^, ^6^

Localised release of antibiotic is imperative for joint replacements as systemic treatment is ineffective due to the limited circulation in the area adjacent to the implant. Incorporating a powdered antibiotic in bone cement has proved to be successful, resulting in a reduction in primary implant infection rates from 7% to lower than 1%.^7^ In a recent survey conducted by the National Joint Registry for England and Wales it was shown that between 2003 and 2010, there has been an increase from 85% to 93% and 92 to 97%, respectively in the percentage of antibiotic‐loaded bone cement used for primary hip and knee replacements.^8^ This trend not only demonstrates the perceived success of this particular technique but also reflects a growing desire to prevent post‐surgical infections in joint replacements. Infected joint replacements are difficult to treat and require the complete removal of the cement and implant. This procedure is costly for both patients and health services due to its complexity, high risk and poor surgical outcome.^9^

Although the use of gentamicin sulfate powder in PMMA bone cement has been successful in reducing the rate of infections in hip and knee replacements, 2,500 revision procedures were still required in the UK in 2012 due to infection^3^ and ∼22,000 revisions of infected hip and knee replacements were performed in the US in 2009.^10^ These figures are projected to increase, as are the costs associated with this complex procedure.^3^, ^10^, ^11^, ^12^ Furthermore, there are concerns that the antibiotic powder may have a detrimental effect on the mechanical properties of the cement. Commercial cements employ 0.5–1 g of gentamicin sulfate per 40 g of cement and this can cause significant reductions in mechanical and fatigue properties of the cement.^13^, ^14^, ^15^, ^16^ The powdered antibiotic is poorly dispersed and agglomerated in the cement and, as a result, only a small percentage of the incorporated gentamicin sulfate is released, the majority in an uncontrolled burst from surface agglomerations within the first few hours.^14^ Sub‐inhibitory concentrations are thought to be released thereafter, which may encourage the establishment of an antibiotic‐resistant bacterial population.^17^

To overcome these problems we propose the use of liposomes to disperse therapeutic agents within bone cement and to control their release characteristics. Liposomes are spherical vesicles composed of lipid molecules that are capable of encapsulating a variety and combination of both hydrophilic and hydrophobic therapeutic agents. The structure, size and composition of the liposome can be tailored for specific applications. Liposomes have been extensively used in medicine for the treatments of infections, cancers, pain management, vaccinations and in ophthalmology.^18^, ^19^, ^20^ Various therapeutic agents have been incorporated within liposomes, including antimicrobial and antineoplastic therapeutic agents, chelating agents, steroids, vaccines and genetic materials.^21^ The majority of these liposomal systems are administered as an aqueous suspension and there is little evidence of their use in polymers or non‐aqueous environments; the hydrophilic head groups on the outermost layer of the liposome are generally not compatible with hydrophobic materials, such as PMMA bone cements. Due to the hydrophobicity of methyl methacrylate, phase separation of the aqueous lipid suspension is likely to occur. For this reason, the use of gentamicin solution in the liquid component of bone cements has also proven unsuccessful as significant reductions in mechanical properties were observed.^22^, ^23^ This was found to occur as a result of the water content and phase separation increasing the size and number of pores. This problem severely limits the range of antibiotics and other drugs that can be incorporated in the cement. To overcome the issue of phase separation, surfactants may be used. Miller et al. attempted to use surfactants with gentamicin solution but found reductions in mechanical properties nevertheless due to the presence of water.^24^ Pelleted liposomes, obtained through ultra‐centrifugation in conjunction with amphiphilic polymers (containing a hydrophobic and a hydrophilic portion) may overcome this issue. Non‐toxic, neutral surfactants, such as Pluronic block copolymers, which improve the miscibility of solutions, may be employed to overcome phase separation of the liposomes in methyl methacrylate. Pluronics consist of two hydrophilic polyethylene oxide (PEO) chains connected by a hydrophobic polypropylene oxide (PPO) chain (PEO‐PPO‐PEO).

The aim of this study was to incorporate gentamicin sulfate into 100 nm extruded liposomes, coated with Pluronics, within the liquid component of a commercial bone cement, Palacos R. The dispersion of the liposomes was studied in methyl methacrylate and within the polymerised cement. Release characteristics, antibacterial activity and mechanical and fatigue properties were evaluated.

## MATERIALS AND METHODS

### Liposome preparation

Phosphatidylcholine (PC) from egg yolk (≥99%), cholesterol (C, ≥99), gentamicin sulfate (≥590 µg gentamicin base per mg) and Pluronics L31 and L61 were purchased from Sigma Aldrich (Sigma‐Aldrich Company Ltd., Gillingham, UK). Pluronic L43 was provided by BASF (BASF Corporation, Connecticut, USA); chloroform (HPLC grade) was purchased from Fisher Scientific (Fisher Scientific UK, Loughborough, UK). 175 mg of PC and 25 mg of C (weight ratio of 7:1) were weighed and dissolved with 5 mL of chloroform in a 50 mL round bottom flask. The chloroform was evaporated using a rotary evaporator at 60°C (above the phase transition temperature of PC) under vacuum. The lipid bilayer was resuspended in 40 mL of 5 mg mL^−1^ gentamicin sulfate solution by vortex mixing. The suspension was then held at 60°C for 30 min prior to 10 extrusions under nitrogen pressure (8 bars maximum) using a Lipex extruder (Northern Lipids, British Columbia, Canada) vertically through a 400 nm polycarbonate membrane (Whatman, UK). The suspension was subsequently extruded a further 10 times through a 100 nm polycarbonate membrane. Average liposome diameter was determined by photon correlation spectroscopy (Beckman Coulter N4 PLUS). L31 Pluronic (2%w/w) was added and the suspension centrifuged at 100,000 g (25,000 RPM) for 1 hr at 4**°**C (Beckman Optima LE‐80K) to create a pellet. The 200‐mg pellet was resuspended in 20 mL of the liquid MMA component of Palacos R) by the process of titruration. The method was repeated for L43 and L61 Pluronics. These Pluronics were employed as they created the most stable liposome suspensions in terms of sedimentation rate and dispersion.^25^

### Transmission electron microscopy

Uranyl acetate (≥98.0%) and methyl methacrylate (≥98.5%) with ≤30 ppm hydroquinone were purchased from Sigma**–**Aldrich (Sigma–Aldrich Company., Gillingham, UK). Transmission electron microscopy (TEM) was used to image the dispersion of liposomes in MMA and water. 100 mg of PC:C liposomes and 100 mg of PC:C liposomes with L61 Pluronic were prepared as described in the Liposome Preparation section at 5 mg mL^−1^ concentration, mixed with an equal volume of 4%w/v aqueous uranyl acetate solution and incubated for 60 min. The liposomes were centrifuged into 20 mg pellets and resuspended into 20 mL of water or MMA. Ten µL of the diluted suspension was placed on a Formvar carbon film on a 400 mesh Nickel grid (EM Systems Support, Macclesfield, UK) and allowed to dry in air. MMA alone was also dried on a grid as a control. Grids were visualised using a Philips CM12 TEM (Philips Research, Eindhoven, Netherlands) operating at 80 kV. Images were recorded using an SIS MegaView III digital camera (Olympus Soft Imaging Solutions GmbH, Münster, Germany).

### Fluorescent microscopy

To assess the dispersion of the liposomes in a commercial cement, 16 mL of a 5 mg mL^−1^ liposome suspension in water was prepared as described in the Liposome Preparation section, however fluorescent TopFluor cholesterol (FC; 23‐(dipyrrometheneboron difluoride)‐24‐norcholesterol; INstruchemie BV, Delfzijl, Netherlands) was used to substitute for a portion of the cholesterol component to give a weight ratio of 7:0.9:0.1 of PC:C:FC. The suspension was divided into four aliquots of 4 mL. A 2%w/w of Pluronic L31, L43, or L61 was added to three of the aliquots and the suspension was centrifuged as previously described. Similarly, the remaining 4‐mL aliquot of the liposomal suspension alone was also centrifuged. The four 20 mg pellets were individually resuspended in 2 mL of the liquid MMA component of Palacos R (Heraeus Medical, Newbury, UK). This was mixed with 4 g of the Palacos R powder as outlined by the ISO5833 guidelines and the cement manufacturer's instructions.^26^ After mixing the cement was compressed between two glass slides to create a thin sample capable of transmitting light. All cement samples were stored in the dark until observed using an Olympus IX50 fluorescent microscope.

### Detection of structural changes using FTIR

Fourier Transform Infrared Spectroscopy (FTIR) was employed to detect changes in the polymer structure due to the presence of the liposomes. Palacos R, Palacos R+G and the liposomal Palacos R cement with L61 Pluronic were scanned using attenuated total reflectance (ATR) with a Perkin Elmer Spectrum One FT‐IR Spectrometer with FT‐IR Spectrum software (Perkin Elmer, Massachusetts, USA) between 4000 and 450 cm^−1^ with a resolution of 4 cm^−1^. The surface of the cement samples were scanned at six different sections on the sample, taking 30 scans at each section to obtain an average spectrum.

### Antibiotic release

O‐phthaldialdehyde (≥97%, HPLC), ethanol (≥99.8%, HPLC), 2‐mercaptoethanol (≥99.0%) and sodium borate (≥99.0%) were purchased from Sigma Aldrich (Sigma–Aldrich Company, Gillingham, UK). Sodium chloride (≥99.9%), potassium chloride (≥99%), calcium chloride (≥99%), and sodium bicarbonate (≥99.7%) were purchased from Fisher Scientific (Fisher Scientific UK, Loughborough, UK). Palacos R and Palacos R+G cements (Heraeus Medical, Newbury, UK) with or without liposomes were mixed as outlined in the manufacturer's instructions and the ISO5833 guidelines.^26^ Table 1 shows the compositions of the cements tested. An ultra‐high molecular weight PTFE mould was manufactured to produce 10 mm diameter by 2‐mm thick cylindrical samples. All samples were abraded with a 250 grit silicon carbide paper to the stated dimensions with a tolerance of ±0.2 mm. Each sample weighed 0.40 ± 0.01 g and five samples for each test group were examined. Each sample was stored in 5 mL of Ringer's solution (8.6 mg mL^−1^ NaCl, 0.3 mg mL^−1^ KCl and 0.33 mg mL^−1^ CaCl_2_, buffered to a pH of 7.4 with NaHCO_3_ 27) at 37**°**C. After 6 hr, 1, 2, 3, 7, 15, 30, and 60 days the Ringer's solution was removed and stored in the dark at −20**°**C before assaying; 5 mL of fresh Ringer's was added as replacement. Ringer's solution was replaced at the selected time points in order to approach sink conditions, a condition where the drug concentration in the eluent can be assumed negligible in comparison to the maximum solubility of the drug (50 mg mL^−1^ for gentamicin sulfate).

**Table 1:** Composition of Palacos R, Palacos R+G, and the Liposomal Palacos R Cements

		Palacos R	Palacos R+G	Liposomal Palacos R (L31/L43/L61)
Liquid	Total liquid g	18.78	18.80	18.80
	Methyl methacrylate %w/w	97.98	97.87	97.88
	*N*,*N* dimethyl‐*p*‐toluidine %w/w	2.02	2.13	2.02
	Hydroquinone ppm	60.00	60.00	60.00
	Liposomal gentamicin sulfate (200mg)/%w/w	–	–	0.11
Powder	Total powder g	40.00	40.80	40.00
	Poly(methyl methacrylate/methyl acrylate) %w/w	84.50	83.27	84.50
	Zirconium dioxide %w/w	15.00	15.00	15.00
	Benzoyl peroxide %w/w	0.50	0.50	0.50
	Gentamicin sulfate (≥590 µg gentamicin base per mg) %w/w	–	1.23	–
Powder: Liquid ratio		2.13	2.17	2.13

The solutions were thawed overnight at room temperature in the dark and the concentration of gentamicin released was determined using an o‐phthaldialdehyde (PHT) method developed by Sampath et al.^28^ and Zhang et al.,^29^ whereby a PHT reagent reacts with the amino groups of gentamicin sulfate to yield measurable fluorogenic products. The reagent was prepared by adding 2.5 g of o‐phthaldialdehyde, 62.5 mL of ethanol and 3 mL of 2‐mercaptoethanol to 560 mL of 0.04M sodium borate solution in distilled water. The PHT reagent was stored in an amber glass bottle in the dark for 24 hr prior to use.

1 mL of the sample eluate was mixed with 1 mL PHT reagent and 1 mL isopropanol and left for 40 min to react and its absorbance compared against a gentamicin calibration curve (ranging from 0 to 100 μg mL^−1^), in order to determine the concentration of gentamicin released by the samples at each time point. Average gentamicin concentrations for each time point were calculated from the five samples and the gentamicin release was determined as a percentage of the theoretical maximum amount of gentamicin sulfate in each sample.

### Mathematical modelling of release

Mathematical models were applied to the antibiotic release data for the commercial bone cements in order to better understand the release kinetics. In the equations described, R_t_ is the total percentage gentamicin sulfate released, t is the time period, n is an exponential function and k is a diffusion related constant. Equation (1) was developed by Korsmeyer et al.^30^ who stated drug release was exponentially related to time.

(1) $$R_{t}{=\;\text{kt}}^{n}$$

Higuchi et al.^31^ proposed Eq. (2), whereby the release of drugs is dependent upon Fickian diffusion of water into the material.

(2) $$R_{t}{=\;\text{kt}}^{1/2}$$

Equation (3) is associated with a Noyes–Whitney dissolution process.^32^ The Noyes–Whitney dissolution process states that the dissolution rate of a solid is dependent on its diffusion coefficient, the surface area of the solid, the concentration at the boundary layer and the length of the boundary layer.

(3) $$R_{t}=\;k\left[1-\text{exp}\left(-\text{nt}\right)\right]$$

Equation (4) is an expanded version of Korsmeyer's work developed by Lindner and Lippold^33^ which takes into account an additional coefficient (b) to represent the initial burst of antibiotics from the surface of the cement. The additional coefficient was added to the Higuchi and Noyes–Whitney equations [Eqs. (1) and (3)] and applied to the release data.

(4) $$R_{t}=b{+\text{kt}}^{n}$$

All models were applied to the data using the curve fitting toolbox in Matlab (MathWorks, Cambridge, MA), which calculates the coefficients by minimizing the sum of squared residuals.

### Agar diffusion assay

Three 10 mm diameter by 2 mm thick cylindrical samples of Palacos R, Palacos R+G, and the liposomal Palacos R cements (L31, L43, and L61) were prepared as previously described in the Liposome Preparation section. *Staphylococcus aureus* (*S. aureus,* NCIMB 9518) was cultured in tryptone soya broth (TSB) for 18–24 hr at 37**°**C. A sterile cotton swab was used to spread the inoculum across the TSA Petri dish. The Petri dish was turned through 60° and the process was repeated to ensure complete surface coverage. A 10 µg gentamicin disc (Oxoid, Hampshire, UK) was placed on the Petri dish as a control and pressure was applied to the top of the disc to ensure full surface contact. The dish was divided into segments and the bone cement samples were individually placed into each segment well separated on the agar, in the same manner. The Petri dish was then incubated at 37°C for 24 hr, after which, the zones of inhibition around the samples and gentamicin disc were measured. Images of the zones of inhibition were taken and analyzed using ImageJ software (National Institutes of Health, MA). The zones of inhibition were measured as the radius of the zone minus the radius of the sample. Two measurements for each zone of inhibition were taken, perpendicular to one another and the experiment was repeated in triplicate (*n* = 3).

### Mechanical properties

The compression and bending properties of Palacos R, Palacos R+G, and the liposomal Palacos R cement samples were determined using a Zwick Roell ProLine table‐top Z050/Z100 materials testing machine with TestXpert II software (Zwick Testing Machines, Herefordshire, UK) following the ISO5833 standard.^26^ Cylindrical compression samples (6 mm diameter and 12 mm length) were tested at a cross‐head speed of 20 mm min^−1^. Rectangular bending samples (75 mm length, 10 mm width, and 3.3 mm thickness) were tested at a cross‐head speed of 5 mm min^−1^ in four‐point bending. Five compression and five bending samples were tested to determine average compression and bending properties.

### Fracture toughness

Linear elastic fracture mechanics (LEFM) was used to determine the fracture toughness of cement samples. Fractured bending samples were employed for single‐edge notched three‐point bending (∼35 mm length, 10 mm width, and 3 mm thickness). A sharp chevron notch (4.5–5.5 mm in length) was created through the center of the sample using a surgical scalpel blade mounted onto a modified microtome, as described by Evans et al.^34^ and the initial crack length and specimen dimensions were measured using a travelling microscope (Pye Scientific, Cambridge, UK). The specimens were loaded at a crosshead speed of 5 mm min^−1^ in three‐point bending, with the span between the rollers set to 40 mm, until failure occurred. The load and displacement were recorded and the critical stress intensity factor (*K*_ic_) of the cement was calculated as described in the ISO13586:2000 standard.^35^ Five samples were tested for each group to produce mean fracture toughness values.

### Glass transition temperature

The glass transition temperature of Palacos R, Palacos R+G, and the liposomal Palacos R cements were determined by dynamic mechanical analysis using a Rheometric Scientific V500 (TA Instruments, Elstree, UK). Small matchstick‐like samples (30 mm length, 3 mm width, and 2 mm height) were prepared and loaded sinusoidally at a frequency of 1 Hz in a dual cantilever configuration whilst heated from 35**°**C to 200**°**C at a rate of 10**°**C min^−1^ with the applied stress and strain measured. The phase lag (*δ*) between the stress (*σ*) and strain (*ε*) was measured and the storage modulus (*E*′) and loss modulus (*E*″) were calculated using Eqs. (5) and (6) respectively. The complex viscosity (tan *δ*) was calculated as shown in Eq. (7) and plotted against temperature. The temperature at which tan *δ* was at its maximum was considered to be the glass transition temperature (*T*_g_). Five samples were tested to obtain a mean value for *T*_g_.

(5) $$E’\;=\;\frac{\sigma }{\varepsilon }\;\text{cos}\;\delta$$

(6) $$E"\;=\;\frac{\sigma }{\varepsilon }\;\sin\;\delta$$

(7) $$\text{tan}\;\delta \;=\;\frac{E\prime }{E\prime \prime }$$

### Vickers hardness

Palacos R, Palacos R+G and the liposomal Palacos R cement disc shaped samples (10 mm diameter and 2 mm height) were prepared and the surfaces polished with a 4000 grit silicon carbide paper. The microhardness of the samples was tested according to the ISO6507‐2 standard.^36^ Five indentations, 1 mm apart and 1 mm from the edges of the sample, were placed on both sides of each sample using a Zwick 3212 indenter fitted with a 136**°** square‐based pyramid diamond indenter (Zwick Testing Machines Ltd., Herefordshire, UK) with a load of 200 g for 10 s. Indentations in the vicinity of large pores (>50 µm) were disregarded and repeated. For each indentation, two diagonal lengths were measured using a travelling microscope and averaged. Equation (8) was employed to calculate the average hardness value (in MPa), where *F* is the load in kg (0.2) and *d* is the mean diagonal length of the indentation in mm.

(8) $$\text{Hardness}\;=\;1.854\;\times \;\frac{F}{d^{2}}$$

### Fatigue

Fatigue crack growth tests were performed using disc compact tension (DCT) specimens, with dimensions conforming to the ASTM E399 standard^37^ as described by Evans et al.^34^ A modified microtome was used to cut a chevron notch, which ensured symmetrical crack growth. The initial crack length and specimen dimensions were measured using a travelling microscope (Pye Scientific, Cambridge, UK). To apply the load symmetrically across the sample, loose plates with oversize holes were employed, which allowed the loading pins to rotate freely without friction. The crack length was monitored using Krak‐gauges and a constant current supply and amplifier designed and built by Evans et al.^34^ was used to obtain accurate crack growth measurements at low crack growth rates. Precision components were employed and the current regulator and preamplifier were kept at a constant temperature in an Instron environmental chamber (Instron SFL, Bristol, UK) to achieve very low noise and offset drift.

Two fatigue samples for Palacos R, Palacos R+G and the liposomal Palacos R cements were tested. All tests were carried out in an Instron environmental chamber at 37°C using a 5 kN Dartec servohydraulic testing machine with an MTS FlexTest GT controller and MTS Multipurpose software (MTS, Eden Prairie, MN).

Samples were cyclically loaded in load control with a sine wave at 5 Hz between 100N and 10N (R‐ratio of 0.1), allowing for measurements of crack growth rates through stress intensities of 0.3 to 0.9 MPam^1/2^. Prior to loading, samples were subject to a precracking procedure to ensure an initial steady crack growth rate of 10^−9^m/cycle. For precracking, a high cyclic load of 200 N at 5 Hz was applied and the load reduced by 10% after every 0.2 mm of crack growth. The crack length and number of cycles were measured during loading and the crack growth rate (da/dN in m/cycle) was calculated for every 0.2 mm of crack growth. Similarly, the corresponding stress intensity factor range (ΔK) for each sample and crack length (ranging from 6 to 12 mm, in steps of 0.2 mm) was calculated using Eq. (9), where *P*_max_ is the maximum load, *P*_min_ is the minimum load, *b* is the sample thickness, a is the crack length from the center point of the holes to the tip of the crack, *W* is the width of the specimen from the center point of the holes to the bottom of the specimen and *α* is *a*/*W* and is >0.2.

(9) $$\Delta K\;=\frac{\left(P_{\text{max}}-P_{\text{min}}\right)}{b\sqrt{w}}\;\times \;\frac{\left(2+\alpha \right)}{{\left(1\;–\;\alpha \right)}^{3/2}}\left(0.76+4.8\alpha -11.58\alpha^{2}+11.43\alpha^{3}-4.08\alpha^{4}\right)\;$$

For each sample, da/dn was plotted as a function of Δ*K* on a logarithmic scale. The Paris Law was applied to obtain coefficients “*A*” and “*m*” [as shown in Eq. (10)] for each sample group.

(10) $$\frac{da}{dn}\;=\;A\;\Delta K^{m}$$

### Scanning electron microscopy

The fracture surfaces of the fatigue testing samples were gold coated using an E65x sputter coater (Emitech, Kent, UK) and imaged using an EBT1 scanning electron microscope (SEM Tech, Southampton, UK) at 15 KeV. Similarly, the surfaces of the antibiotic release samples were imaged to study surface porosity.

### Statistical analysis

An analysis of variance (ANOVA) was carried out to establish significant differences between groups of samples using the data analysis package in Excel (Microsoft, Reading, UK). Significance between groups was defined as those with a calculated *p* values of <0.05.

## RESULTS

### Liposome diameter (with and without Pluronics in water)

Prior to extrusion the average diameter of the liposomes was 588 ± 243 nm. After 10 extrusions through a 400 nm polycarbonate membrane and 10 extrusions through a 100 nm polycarbonate membrane, the average diameter of the liposomes was 100 ± 19 nm. After ultra‐centrifugation and resuspension, the diameter was 111 ± 16 nm. The addition of Pluronics L31, L43, and L61 did not alter the average diameter of the liposomes (108 ± 28 nm, 106 ± 21 nm, and 101 ± 30 nm, respectively).

### Transmission electron microscopy (TEM)

**Figure 1:**
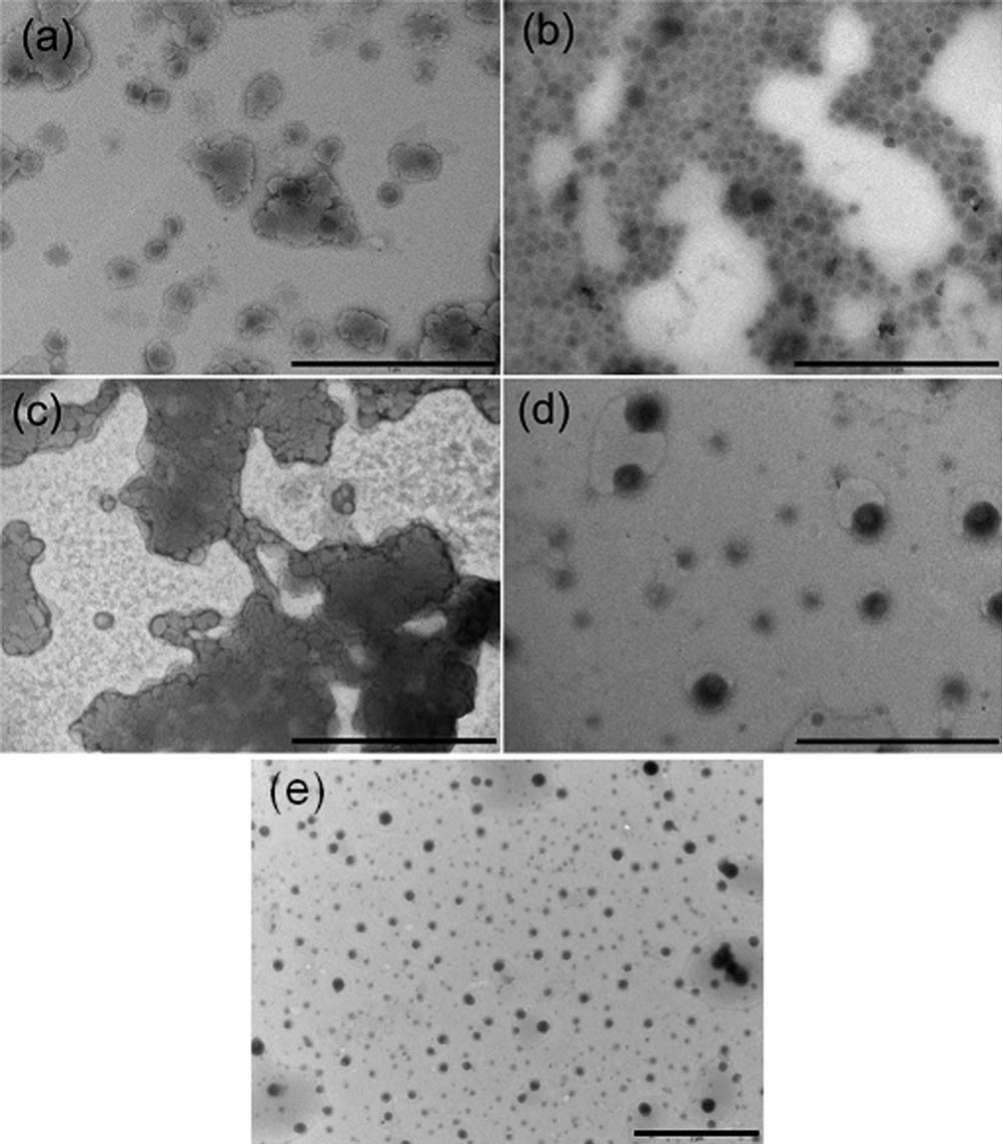
TEM images of liposomes in (a) water and (b) MMA and liposomes with Pluronic L61 in (c) water, (d) MMA (Bar = 1 µm) and (e) liposomes with Pluronics L61 in MMA at a lower magnification.

Figure 1 shows TEM images of liposomes with and without the addition of Pluronic L61 resuspended in water or MMA. After centrifugation and resuspension the individual liposomes were intact and approximately 100 nm in diameter, demonstrating that centrifugation at 100,000*g* for 1 hr did not damage the lipid bilayer and confirming the results obtained by photon correlation spectroscopy. Small agglomerations were observed when liposomes were resuspended in water after centrifugation [Figure 1(a)], whilst large agglomerations were observed when the liposomes with Pluronic L61 were resuspended in water [Figure 1(b)]. When resuspending the liposomes in MMA without Pluronic L61, the liposomes were found to coalesce potentially due to the hydrophilic interactions between the polar heads of the phospholipids in the hydrophobic MMA [Figure 1(c)]. Improved dispersion was achieved however when liposomes with Pluronic L61 were resuspended in MMA [Figure 1(d)]. Individual liposome diameters were **∼**100 to 200 nm, confirming the results observed by photon correlation spectroscopy.

### Fluorescence microscopy

**Figure 2:**
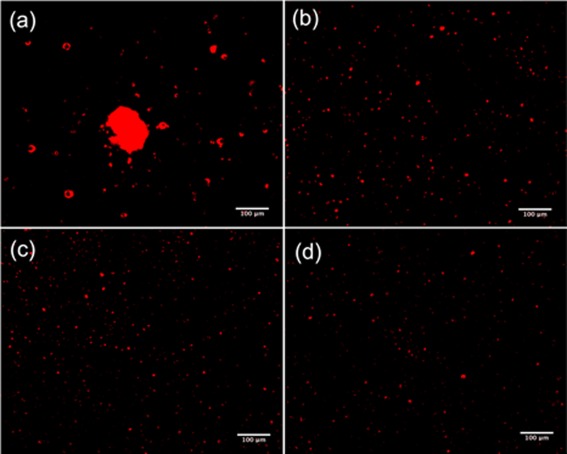
Fluorescence microscopy images of fluorescent liposomes (a) without coating and with Pluronics (b) L31, (c) L43, and (d) L61 in Palacos R cement (Bar = 100 µm). [Color figure can be viewed in the online issue, which is available at wileyonlinelibrary.com.]

The fluorescent liposome pellets were resuspended in the liquid component of Palacos R bone cement prior to combination with the powder component and polymerization. Figure 2 shows fluorescent microscopy images of the cement surfaces containing liposomes with and without Pluronics L31, L43, and L61. Similar results to the TEM images were observed; when resuspending liposomes without Pluronics in Palacos R cement, high levels of agglomeration were observed [Figure 2(a)]. The Pluronics were found to enhance the dispersion of the liposomes throughout the cement [Figure 2(b–d)].

### Detection of structural changes using FTIR

**Figure 3:**
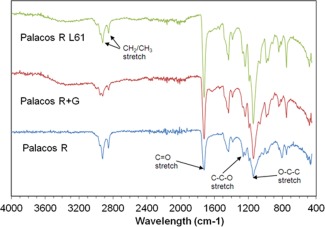
FTIR spectra of Palacos R, Palacos R+G and liposomal Palacos R with Pluronic L61 using attenuated total reflectance (ATR).

FTIR was employed to detect potential changes in polymerization or cement structure arising from incorporating liposomes. Figure 3 shows the FTIR spectra for Palacos R, Palacos R+G and liposomal Palacos R with L61 Pluronic using attenuated total reflectance. FTIR peaks representative of PMMA were observed in all samples. These peaks were: between 2850 and 2950 cm^−1^ for methylene (CH_2_) and methyl (CH_3_) stretches; between 1300 and 1500 cm^−1^ for the CH bend; at 1400 and 1450 cm^−1^ for methyl groups (CH_3_); at 1240 cm^−1^ for the C—C—O stretch; at 1140 cm^−1^ for the O—C—C stretch; at 1730 cm^−1^ for the C=O stretch; and between 1300 and 900 cm^−1^ for the C—O stretch. Broad bands were observed at **∼**3448 cm**^−^** ^1^ and sharp bands at 1636 and 1051 cm**^−^** ^1^ for Palacos R+G and Palacos R with liposomes due to the presence of gentamicin sulfate. No other differences were observed between all three cements, demonstrating the liposomes did not interact or alter the polymerization or structure of Palacos R.

### Antibiotic release

**Figure 4:**
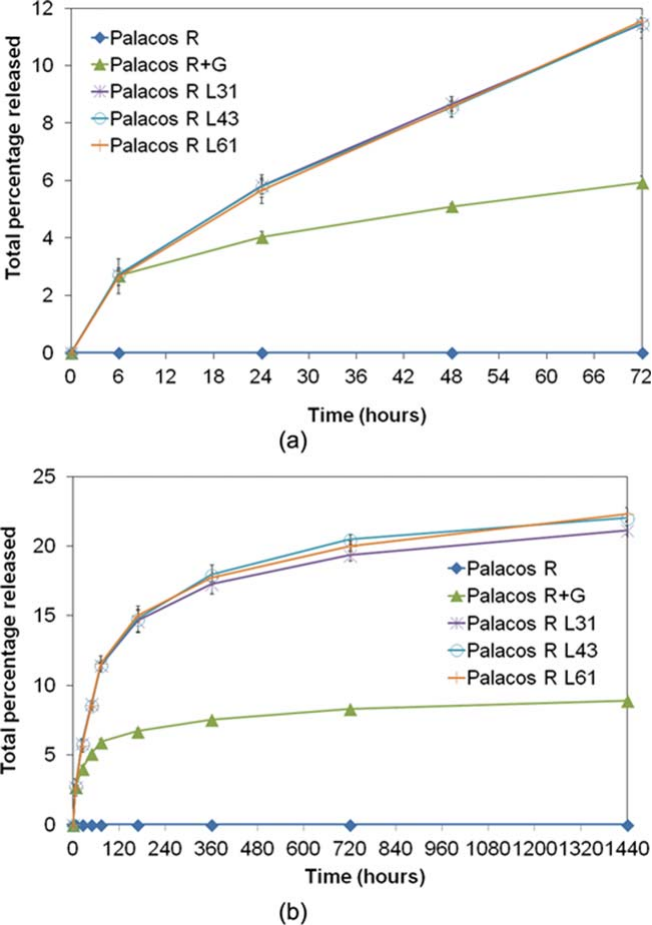
Total percentage gentamicin sulfate release for Palacos R, Palacos R+G and the liposomal Palacos R cements over (a) 72 hr and (b) 1440 hr.

A strong linear correlation was obtained between gentamicin sulfate concentration and absorbance at 340 nm using the o‐phthaldialdehyde method (*R* value of 0.9987), with acceptable standard deviations up to gentamicin sulfate concentrations of 90 μg mL^−1^ (absorbance of 1.6). The percentage antibiotic release from Palacos R, Palacos R+G and the liposomal Palacos R cements with L31, L43, and L61 Pluronics after 72 hr and 1440 hr are shown in Figure 4(a,b), respectively. The liposomal Palacos R cements performed more efficiently than the Palacos R+G. At 6 hr, a similar percentage release was experienced by Palacos R+G and the liposomal Palacos R cements (≃2.7%), however after this time the liposomal Palacos R cements achieved much higher percentages. At 72 hr, the liposomal Palacos R released 11–12% of the total gentamicin sulfate used, whilst Palacos R+G released **∼**6%. Over the first 72 hr gentamicin release from the liposomal Palacos R cements was more linear than the Palacos R+G, which experienced an initial burst of release followed by lower levels of release thereafter [Figure 4(a)]. Furthermore, the standard deviations experienced for all liposomal Palacos R cements within the first 72 hr were less than those of the commercial Palacos R+G cement containing powdered gentamicin. After 1440 hr, the liposomal Palacos R cements had released 21‐22% of the incorporated gentamicin, more than Palacos R+G **[**8.9%, Figure 4(b)].

### Mathematical modelling of release

The results from the mathematical modelling of the gentamicin sulfate release from Palacos R+G and the liposomal Palacos R cement are shown Table 2. Coefficient values (*k*, *n*, and *b*) that most accurately model the experimental antibiotic release data are shown. The table also shows the goodness of fit (*R* value) between the experimental data and the models.

**Table 2:** Mathematical Modelling Results of Gentamicin Sulfate Release for Palacos R+G and the Liposomal Palacos R Cements

		Korsmeyer; *R*_t_ = kt^n^	Higuchi; *R*_t_ = kt^1/2^	Noyes–Whitney; *R*_t_ = k[1 − exp(−nt)]	Expanded Korsmeyer; *R*_t_ = *b* + kt^n^	Expanded Higuchi; *R*_t_ = *b* + kt^1/2^	Expanded Noyes–Whitney; *R*_t_ = *b* + *k*[1 − exp(−nt)]
Palacos R+G	*k*	2.421	0.696	7.827	24.487	0.160	5.541
	*n*	0.284	0.500	0.011	0.039	0.500	0.010
	*b*	–	–	–	−23.426	3.764	2.744
	*R* value	0.9559	−0.4704	0.8204	0.9885	0.8337	0.9570
Palacos R L31	*k*	3.142	0.716	19.070	56.050	0.496	16.930
	*n*	0.273	0.500	0.012	0.051	0.500	0.008
	*b*	–	–	–	−58.930	5.224	2.698
	*R* value	0.9345	0.5828	0.9555	0.9855	0.8483	0.9761
Palacos R L43	*k*	3.039	0.743	20.072	48.389	0.531	17.830
	*n*	0.284	0.500	0.011	0.060	0.500	0.007
	*b*	–	–	–	−51.675	5.045	2.938
	*R* value	0.9395	0.6420	0.9559	0.9857	0.8612	0.9796
Palacos R L61	*k*	2.861	0.696	18.746	74.105	0.498	17.324
	*n*	0.284	0.500	0.011	0.040	0.500	0.009
	*b*	–	–	–	−77.852	4.700	1.736
	*R* value	0.9187	0.6242	0.9716	0.9797	0.8334	0.9803

For all cements, the n‐coefficient for the Korsmeyer–Peppas model was found to be <0.5, demonstrating that the release of gentamicin sulfate from the cements was not governed by Fickian diffusion alone. The Higuchi model for the liposomal Palacos R cements however had higher R‐values than Palacos R+G demonstrating release from the liposomal cements is more related to diffusion‐based mechanisms. The Korsmeyer–Peppas model was found to best model the Palacos R+G release profile, whilst the Noyes‐Whitney dissolution model fitted the experimental release data for the liposomal Palacos R cements the best. The *R* values obtained from the Noyes–Whitney and the expanded Noyes‐Whitney models were higher for the liposomal Palacos R cements than the Palacos R+G cements. This demonstrates a more dissolution‐based process taking place in the liposomal Palacos R cements. The additional coefficient (b) was found to improve the accuracy of the mathematical models for all the cements tested confirming there was a burst of antibiotic from the surface of the cement. Interestingly, the burst coefficient improved the models for Palacos R+G to a greater extent than the liposomal Palacos R cements.

### Agar diffusion assay

**Figure 5:**
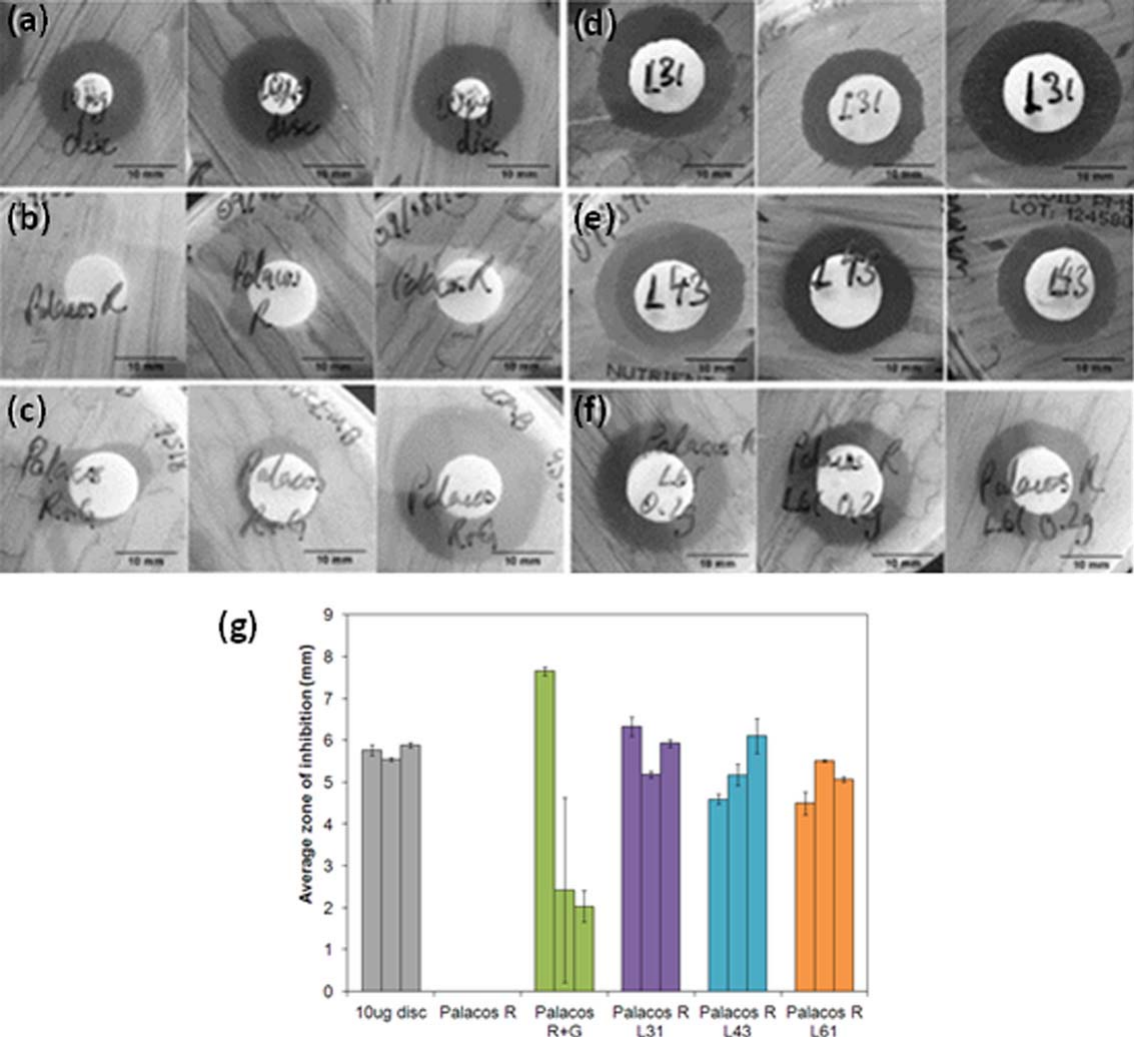
Zones of inhibition for *S. aureus* on tryptone soya agar. *S. aureus* was susceptible to 10 µg of gentamicin (a), whilst Palacos R alone was not antimicrobial (b). Palacos R+G demonstrated inconsistent zones of inhibition (c), whilst the liposomal Palacos R cements consistently inhibited S. aureus growth around the cement (d–f).

Figure 5 shows the zones of inhibition obtained from the agar diffusion assay and the average radii of the zones. No zones of inhibition were obtained for Palacos R cement, demonstrating no antimicrobial activity against *S. aureus* [Figure 5(b)]. Palacos R+G was found to produce the largest zone of inhibition in one sample, however the variation between samples was large, potentially due to variable dispersion of the gentamicin sulfate in the cement and/or poor release properties [Figure 5(c)]. The liposomal Palacos R cements (L31, L43, and L61) however produced consistent inhibition zones demonstrating reliable antimicrobial activity against *S. aureus* [Figure 5(d–f)]. Furthermore, these results highlight the availability of the encapsulated gentamicin sulfate to inhibit *S. aureus* growth.

### Mechanical properties, fracture toughness, glass transition temperature and hardness

The mechanical properties, fracture toughness, glass transition temperature and hardness of Palacos R, Palacos R+G and the liposomal Palacos cements with L31, L43, and L61 Pluronics are shown in Table 3. Palacos R+G had a significantly lower compressive strength, bending modulus, bending strength and glass transition temperature when compared to Palacos R cement. This may be due to the weakening effect of the gentamicin sulfate particles. The liposomal Palacos R cements all experienced a significant reduction in compressive strength when compared to both Palacos R and Palacos R+G cements. Interestingly, the bending strength, fracture toughness and Vickers hardness of all the liposomal Palacos R cements were significantly improved when compared to the Palacos R and Palacos R+G cement samples. This demonstrates that the well‐dispersed sub‐micron liposomes are having a positive effect on the toughness of the cement. Similarly, the liposomes were found to increase the glass transition temperature of the cement.

**Table 3:** Mechanical Properties and Fracture Toughness for Palacos R, Palacos R+G, and the Liposomal Palacos Cements with L31, L43, and L61 Pluronics

	Compressive Strength (MPa)	Bending Strength (MPa)	Bending Modulus (MPa)	Fracture Toughness (MPam^1/2^)	Glass Transition Temperature (°C)	Vickers Hardness (MPa)
Palacos R	104.6 ± 2.6	73.4 ± 2.9	3460 ± 97	2.5 ± 0.2	126.2 ± 3.3	19.8 ± 1.8
Palacos R+G	95.7 ± 3.3^a^	66.6 ± 2.4^a^	3110 ± 110^a^	2.5 ± 0.1	115.4 ± 1.2^a^	18.7 ± 1.8
Palacos R L31	80.8 ± 3.4^a^, ^b^	79.0 ± 3.9^b^	3200 ± 61^a^	3.0 ± 0.3^a^, ^b^	122.7 ± 2.2^b^	26.6 ± 3.2^a^, ^b^
Palacos R L43	77.4 ± 2.4^a^, ^b^	73.0 ± 4.3^b^	3330 ± 181	2.9 ± 0.2^a^, ^b^	121.8 ± 2.1^b^	25.9 ± 1.9^a^, ^b^
Palacos R L61	78.2 ± 1.5^a^, ^b^	74.1 ± 3.4^b^	3270 ± 137	2.9 ± 0.2^a^, ^b^	123.2 ± 1.4^b^	28.1 ± 3.8^a^, ^b^

a Significantly different from Palacos R.

b Significantly different from Palacos R+G, ANOVA *p* < 0.05).

### Fatigue

**Figure 6:**
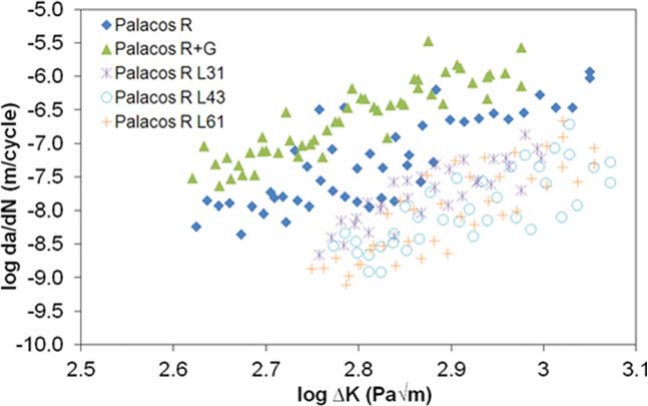
Fatigue crack propagation test results for Palacos R, Palacos R+G and the liposomal Palacos R cements.

Figure 6 shows the fatigue crack propagation results for Palacos R, Palacos R+G and the liposomal Palacos R cements with L31, L43, and L61 Pluronics and Table 4 shows the fatigue test constants obtained when the Paris law was applied to the fatigue data. All three liposomal Palacos R cements performed similarly in terms of crack growth rates. Palacos R+G had a higher rate of crack growth when compared to Palacos R, demonstrating the addition of gentamicin sulfate powder to bone cement reduces the crack resistance of the cement. Interestingly, the addition of well‐dispersed 100 nm liposomes to the cement increased the fatigue crack growth resistance of the cement. Higher stress intensities were required to propagate crack growth in the liposomal cement samples and lower rates of crack growth (da/dN) were observed. When applying the Paris law to the fatigue data, the variables ‘A’ and “*m*” are obtained, where “*A*” is the crack growth rate at a stress intensity of 1 MPam^1/2^ and “*m*” is the slope of the plot. The rate of crack growth at 1 MPam^1/2^ was increased as a result of using gentamicin sulfate powder in the cement, however the use of sub‐micron liposomes was found to reduce the crack growth rates of the cement.

**Table 4:** Fatigue Test Constants Obtained When Applying the Paris Law to the Fatigue Data, Where *A* is the Crack Growth Rate at 1 MPam^1/2^ and *m* is the Slope of the Plot

	*A*	*m*	Correlation Coefficient
Palacos R	3.73 × 10^−07^	4.65	0.67
Palacos R+G	2.62 × 10^−06^	5.01	0.81
Palacos R L31	8.50 × 10^−08^	5.39	0.72
Palacos R L43	2.68 × 10^−08^	4.85	0.61
Palacos R L61	4.45 × 10^−08^	6.47	0.72

### Scanning electron microscopy

**Figure 7:**
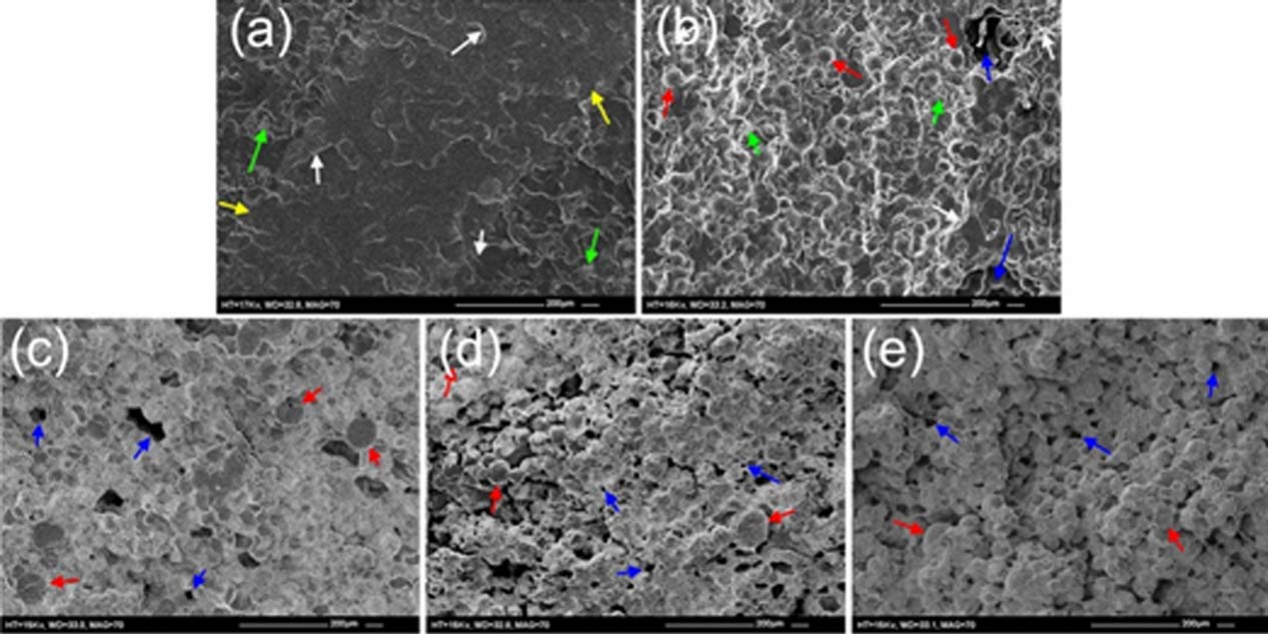
SEM images of the fatigue fracture surfaces for (a) Palacos R, (b) Palacos R+G and liposomal Palacos R with Pluronics (c) L31, (d) and (e) L61. Arrows indicate PMMA beads, pores and microcracks.

Figure 7 shows representative SEM images of the fracture surfaces of the fatigue samples of Palacos R [Figure 7(a)], Palacos R+G [Figure 7(b)] and the liposomal Palacos R cements with L31, L43 and L61 Pluronics [Figure 7(c–e), respectively]. All cements were found to have porosity due to air entrapped during mixing and cleaving of PMMA beads during fracture. The liposomal Palacos R cements were found to have smaller sized pores (20–50 μm), whilst Palacos R+G had larger pores (50–100 μm). Closer inspection of the liposomal Palacos R cements found small well‐dispersed pores (<10 μm) throughout the fracture surface. Rough fracture surfaces were also observed for the liposomal Palacos R cements, indicating non‐brittle fracture.

**Figure 8:**
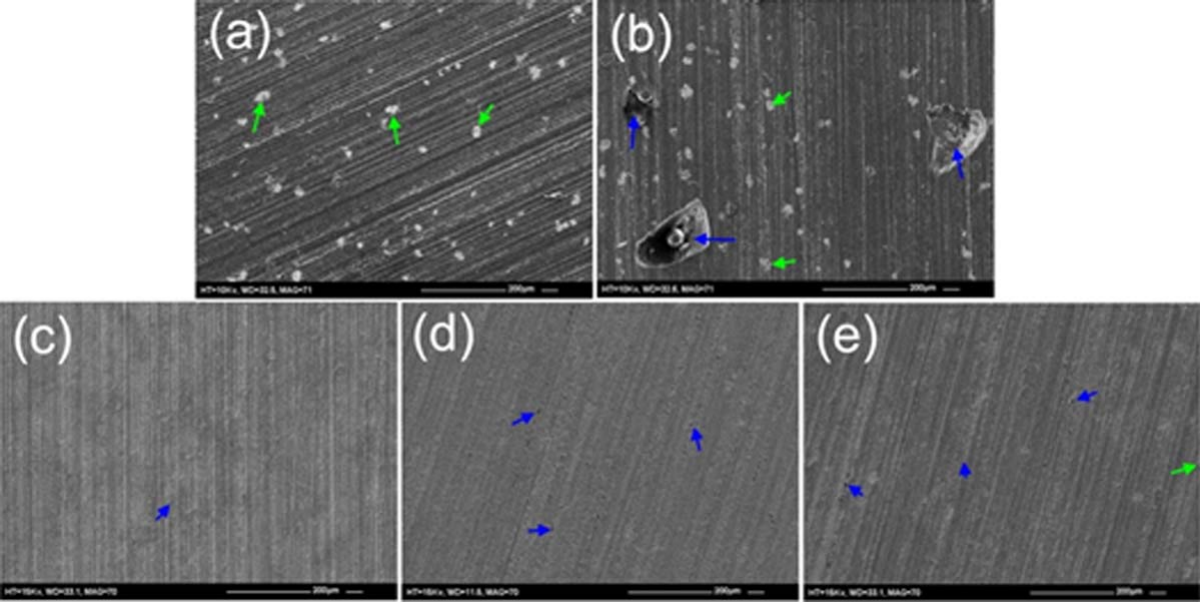
Surface SEM images of the antibiotic release samples after 1440 hr incubation for (a) Palacos R, (b) Palacos R+G and liposomal Palacos R with Pluronics (c) L31, (d) L43 and (e) L61. Arrows indicate radiopacifier agglomerations and pores.

SEM images of the surface of the samples used at the end of the antibiotic release study (Figure 8) showed that Palacos R+G had 50–200 μm pores on the surface of the cement [Figure 8(b)]. Close examination of the surface of the liposomal Palacos R cements revealed small well‐dispersed pores (<10 μm), as found in the fracture surfaces [Figure 8(c–e)].

## DISCUSSION

Commercially available antibiotic‐loaded bone cements, such as Palacos R+G, employ large amounts of gentamicin sulfate powder to achieve therapeutic levels of release (from 0.5 to 1 g per 40 g of cement). Although these cements have reduced infection rates in cemented joint replacements, there are several limitations associated with this delivery system, as shown in this study. A large dose of the antibiotic is released in an uncontrolled manner from the surface of the cement within the first 6 hr, after which, lower, possibly sub‐inhibitory levels, are released over prolonged periods of time. This may promote the evolution of resistant bacteria.^17^ Although large amounts of gentamicin sulfate are incorporated in commercial cements, only a small percentage (∼9% over 60 days) is released, leaving the majority of the antibiotic unused in the bulk of the cement. Large amounts of powdered gentamicin in bone cements have been shown to affect mechanical and fatigue properties in previous studies,^13^, ^14^, ^15^, ^16^ with no beneficial influence against the formation of biofilms.^38^, ^39^ Furthermore, the poor dispersion of gentamicin sulfate in bone cement can result in poor antimicrobial activity. With these limitations in mind, there is a clear requirement to improve antibiotic‐loaded cements.

**Figure 9:**
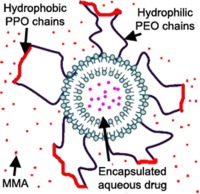
Proposed liposome‐Pluronics structure.

This study demonstrates that liposomes with an encapsulated antibiotic can be effectively dispersed in PMMA bone cement using specific Pluronics (L31, L43, and L61) as surfactants. It is hypothesized that the hydrophilic PEO chains of the Pluronics attach to the outer hydrophilic polar head groups of the liposome leaving the hydrophobic PPO chain to interact with a hydrophobic environment, as shown in Figure 9. This in turn allows the hydrophilic liposomes to be suspended in a non‐aqueous environment such as methyl methacrylate. TEM and fluorescent microscopy showed that in the absence of Pluronics, the liposomes aggregated in the cement, likely due to the hydrophilic outer surfaces of the liposomes attracting one another in a hydrophobic environment. The presence of Pluronics on the surface of the liposomes is thought to render the liposome hydrophobic, enhancing dispersion in non‐aqueous environments. When testing various Pluronic–liposome combinations, it was observed that hydrophobic Pluronics (with a long PPO chain) and a low molecular weight (such as Pluronics L31, L43, and L61) produced the most stable suspensions.^25^ This resulted in an even dispersion of liposomes throughout the cement. All three Pluronics gave similar results in terms of antibiotic release, antimicrobial efficacy and mechanical, fatigue and material properties as a result of good dispersion properties.

For the liposomal Palacos R cements, there was a small initial burst of gentamicin sulfate released from the cement as surface liposomes came into contact with water. The initial burst of gentamicin sulfate was lower than for the commercial cements as significantly less gentamicin sulfate was employed and fewer agglomerations were present on the surface. This resulted in a more linear, gradual and prolonged release of antibiotic over time from the liposomal systems. The SEM images of the release samples confirm this as the commercial cements had randomly located large pores caused by the release of surface agglomerations of gentamicin sulfate, whilst the liposomal Palacos R cements had much smaller well‐dispersed pores. The presence of small well‐dispersed pores would promote controlled diffusion of water through the outer layers of the cement. This in turn would allow water to penetrate uniformly to the antibiotic below the surface of the cement, resulting in a more gradual and controlled release; a previous study has demonstrated PMMA bone cement to absorb moisture over 60 days.^40^ Large random pores caused by agglomerations of gentamicin sulfate powder would result in less controlled release as water penetration into the cement would be less uniform. The standard deviations for the antibiotic release from liposomal Palacos R cements were also reduced, demonstrating consistency between the samples tested over the early time points. Commercial cements on the other hand demonstrated large standard deviations presumably due to variability in the levels of antibiotic being released from each sample.

After 360 hr, the release from the liposomal Palacos R cements was equal to that of Palacos R+G, despite there being 60% less gentamicin sulfate in the liposomal Palacos R cements. This resulted in a much higher percentage release (11‐12% at 72 hr and 21–22% at 1440 hr); more than double that of Palacos R+G. A recent study using liposomal amphotericin B in Simplex P bone cement obtained similar improvements over cements with powdered amphotericin B deoxycholate.^41^

Static release tests were carried out to compare the release from liposomal Palacos R cements with the release from Palacos R+G. Although dynamic elution tests would more accurately replicate *in vivo* conditions, the static release test used provides valuable comparative information on the release of gentamicin sulfate as a result of water penetration. Results obtained using dynamic elution techniques would subject the cement to a continuous circulation of medium, which may encourage degradation of the cement, allowing for increased water penetration and antibiotic release. Although Ringer's solution at 37°C replicated similar temperatures, pH and salt concentrations found *in vivo*, the viscosity of the solution differs from blood, which is increased due to the presence of plasma and particles, such as red blood cells. *In vivo* therefore, the penetration of blood into the cement may differ from that of Ringer's solution, resulting in different release rates.

Centrifugation of liposomes may not harvest all the lipid material for inclusion in the MMA, but calculations on gentamicin content have assumed that all the liposomes were incorporated in cement. Furthermore, a high encapsulation efficiency (nearing 100%) of gentamicin sulfate was also assumed, however studies have shown the encapsulation efficiency of gentamicin sulfate in liposomes to range from 5 to 40%.^42^, ^43^ Therefore, the efficiency of gentamicin sulfate release from the liposomal Palacos R cement may be significantly greater than the calculated results.

The high efficiency of delivery from the liposomal system may be attributed to the release mechanisms taking place. Equations for antibiotic release were used in an attempt to establish this mechanism. Although simplified equations were employed, it was possible to establish the dominant release mechanism in the cements tested. It is likely; however, that a combination of mechanisms are occurring during the release of gentamicin sulfate and therefore, combining release equations may provide more accurate models, as demonstrated by Kuhn and Wilson.^44^ The results from the mathematical models have highlighted differences between the liposomal Palacos R cements and Palacos R+G. The poor fit for the Higuchi model highlights that antibiotic release from bone cement is governed by more than simple diffusion mechanisms. The release from liposomal cements are thought to be controlled through a more diffusion and dissolution based process when compared to Palacos R+G, most likely because the well‐dispersed liposomes create a more controlled pathway for water to penetrate into the cement. All cements experienced a burst of release from surface‐held antibiotic as demonstrated by the improved fit in the expanded models. This improvement was more dramatic for Palacos R+G, demonstrating Palacos R+G has a greater reliance on surface‐driven release mechanism.

The agar diffusion test demonstrated good bioavailability of the encapsulated gentamicin sulfate in the liposomal Palacos R cements as reproducible zones of inhibition against *S. aureus* were obtained, consistent in both size and shape between all the liposomal Palacos R samples. Good dispersion of the liposomes on the surface layers of cement would result in an even diffusion of gentamicin sulfate through the agar, creating consistent zones of inhibition. It can be concluded, therefore, that the liposomal system would most likely inhibit bacteria in all regions of the cement, unlike powdered antibiotic based products which were found to have inconsistent zones of inhibition due to an uneven distribution of gentamicin sulfate. The efficacy of the liposomal system was only tested against one strain of bacteria as a bioindicator. A study by Mugabe et al.^45^ has shown liposomal delivery of therapeutics to be more effective against bacteria than free drugs. The mechanism of action of the liposomes was shown to be through fusion to the wall of the bacterium. This not only enhanced the antimicrobial activity of the encapsulated therapeutic but also increased drug penetration and delivery, making it an exciting method to combat infections caused by antibiotic‐resistant bacteria.

Although the liposomal Palacos R cements were found to have good release and antimicrobial properties, a reduction in compressive strength was experienced although this still remained within the range specified by international standards. It is thought that having a dispersion of sub‐micron liposomes in the cement promotes shear yielding, which occurs near the surface of the material where it is in plane stress. This reduces the compressive strength but increases the toughness of the material as observed. A study using lyophilized liposomes in bone cement also found a similar reduction in compressive strength.^41^ The bending modulus of the liposomal Palacos R cement was not significantly different from Palacos R+G. Improvements in bending strength were observed for the liposomal Palacos R cement when compared to Palacos R+G. This is due to the even dispersion of the liposomes which contrasts with the weakening effect of agglomerations of powdered gentamicin sulfate. No improvements in bending strength however were observed when compared to Palacos R cement.

The compression and bending tests were carried out according to the ISO5833 standard, which all cements must undergo prior to approval for medical application. The liposomal Palacos R cements were found to exceed the ISO5833 minimum requirements for compression and bending properties, demonstrating the novel delivery system retains the suitability of Palacos cement as an acrylic cement for orthopaedics.

After the release of gentamicin sulfate from the liposomal Palacos R cements, small well‐dispersed pores were observed in the SEM images. It has been hypothesised that small dispersed pores may be beneficial to mechanical and fatigue properties by promoting shear yielding ahead of the crack tip, as well as blunting the crack tip.^46^, ^47^ Small pores are also thought to reduce polymerization shrinkage, reducing the likelihood of residual stress‐induced cracks.^48^ It is postulated that the sub‐micron pores and liposomes in the liposomal Palacos R cements behave similar to small rubber particles, inducing toughening mechanisms. Rubber particles have been shown to improve the fracture toughness of bone cement through shear yielding, crack deviation and craze formation,^49^, ^50^, ^51^ but reduce compressive strength as yielding occurs in compression.^52^, ^53^ Furthermore, rubber particles have also been shown to reduce the modulus of materials, as experienced with the liposomal Palacos R cements.^54^ Toughening through shear yielding mechanisms in the liposomal Palacos R cements was observed due to an increase in fracture toughness and fatigue crack propagation. The fracture surfaces were also found to be rougher in the liposomal Palacos R cements, demonstrating non‐brittle fracture.

Tests on the structure of liposomal Palacos R cements demonstrated that liposomes do not affect the molecular structure of the cement; however, the hardness of the cement was significantly increased by the addition of liposomes. As hardness value is linked to the yield stress and toughness of a material, it is expected that the yield stress of the liposomal Palacos R cements would also be significantly higher than Palacos R/R+G. The glass transition temperature was also affected, however at temperatures well below the *T*_g_ of the cements (for example, *in vivo* temperatures of 37°C), it is expected that the slight change will not be detrimental to the mechanical properties as fatigue testing at 37°C was not detrimental to crack growth rates. The ISO5833 standard used for mechanical testing does not specify required testing temperatures. Nevertheless, testing at this temperature would ensure that the changes in *T*_g_ experienced do not significantly affect the compression and bending properties of the liposomal Palacos R cements. Furthermore, creep testing would provide useful information on the rate of deformation that the novel cement is likely to undergo *in vivo*.

## CONCLUSION

Liposomes have been successfully incorporated into a commercial PMMA bone cement (Palacos R) and, with the exception of compressive strength, the commercial cement properties were not adversely affected. Furthermore, all cement properties were above the ISO5833 minimum requirements for acrylic resin cements and the presence of well‐dispersed liposomes throughout the cement improved the bending, fracture toughness, fatigue and hardness properties of the commercial cement. It is speculated that the improvement in fracture toughness and fatigue crack growth resistance is caused by the dispersed liposomes inducing rubber toughening mechanisms, such as shear yielding. The novel drug delivery system demonstrated enhanced dispersion of antibiotics when compared to the current powdered antibiotic system. This resulted in a more controlled, gradual and prolonged release of gentamicin sulfate; more consistent antimicrobial properties and higher efficiency, requiring less gentamicin sulfate to achieve therapeutic levels of release. It is speculated that the liposome dispersion allows water to penetrate and diffuse below the surface of cement in a controlled manner.

Although the initial results are promising, it will now be essential to study the long‐term stability of liposomes in MMA and determine how liposome modified bone cements perform *in vivo*. The system must also be optimised in terms of the antibiotics used; in this regard the ability to employ antibiotics in aqueous solution may broaden the range of agents finding application. Further optimization will include: the size and composition of the liposomes; the encapsulation efficiency and the use of other preparation methods, such as lyophilization, to ensure optimum release, antimicrobial, mechanical and fatigue properties *in vivo*.

In conclusion, the release of liposomal therapeutic agents from PMMA bone cement may play a vital role in reducing infection rates and potentially improving osseointegration in cemented joint replacements and therefore enhance patient quality of life, whilst reducing costs for healthcare providers.

## References

[1] Lewis G. Properties of acrylic bone cement: State of the art review. J Biomed Mater Res 1997;38:155–182. 9178743 10.1002/(sici)1097-4636(199722)38:2<155::aid-jbm10>3.0.co;2-c

[2] Whitehouse MR , Evans SL. Bone cement: An overview. Int J Nano Biomater 2010;3:4–19.

[3] UK NJR. National Joint Registry for England and Wales, 9th Annual Report 2012 Hertfordshire: National Joint Registry UK; 2013. Available at: http://www.njrcentre.org.uk/njrcentre/Portals/0/Documents/England/Reports/10th_annual_report/NJR%2010th%20Annual%20Report%202013%20B.pdf [accessed 9th March 2015]

[4] Buchholz HW , Engelbrecht H. Uber die Depotwirkung einiger Antibiotica bei Vermischung mit dem Kunstharz Palacos. Chirurg 1970;41:511–515. 5487941

[5] Whelton A. The aminoglycosides. Clin Orthop 1984;190:66–74. 6386262

[6] Wahlig H , Dingeldein E. Antibiotics and bone cements: Experimental and clinical long‐term observations. Acta Orthop Scand 1980;51:49–56. 7376844 10.3109/17453678008990768

[7] Buchholz HW , Elson RA , Lodenkämper H. The infected joint implant. In: Recent advances in Orthopaedics 3 B McKibbin, editor. New York: Curchhill Livingston; 1979 pp 139–161.

[8] UK NJR. National Joint Registry for England and Wales, 8th Annual Report 2011. Hertfordshire: National Joint Registry UK, 2011. Available at: http://www.njrcentre.org.uk/NjrCentre/Portals/0/Documents/NJR%208th%20Annual%20Report%202011.pdf. [accessed 9th March 2015]

[9] Cherubino P , Puricelli M , D'Angelo F. Revision in cemented and cementless infected hip arthroplasty. Open Orthopaed J 2013;7:190–196. 10.2174/1874325001307010190 PMC3722547 23898351

[10] Kurtz SM , Lau E , Watson H , Schmier JK , Parvizi J. Economic burden of periprosthetic joint infection in the United States. J Arthroplasty 2012;27 (Suppl 8):61–5.e1. 22554729 10.1016/j.arth.2012.02.022

[11] Kurtz SM , Lau E , Schmier J , Ong KL , Zhao K , Parvizi J. Infection burden for hip and knee arthroplasty in the United States. J Arthroplasty 2008;23:984–991. 18534466 10.1016/j.arth.2007.10.017

[12] Briggs T. Getting it right the first time: Improving the quality of orthopaedic care within the NHS in England. 2012. Available at: http://www.gettingitrightfirsttime.com/downloads/BriggsReportA4_FIN.pdf. [accessed 2nd March 2015]

[13] Dunne N , Hill J , McAfee P , Todd K , Kirkpatrick R , Tunney M , Patrick S . *In vitro* study of the efficacy of acrylic bone cement loaded with supplementary amounts of gentamicin: Effect on mechanical properties, antibiotic release, and biofilm formation. Acta Orthopaed 2007;78:774–785. 10.1080/17453670710014545 18236183

[14] Dunne N , Hill J , McAfee P , Kirkpatrick R , Patrick S , Tunney M. Incorporation of large amounts of gentamicin sulphate into acrylic bone cement: Effect on handling and mechanical properties, antibiotic release, and biofilm formation. Proc Inst Mech Eng H 2008;222:355–365. 18491704 10.1243/09544119JEIM355

[15] Lautenschlager EP , Jacobs JJ , Marshall GW , Meyer PR. Mechanical properties of bone cements containing large doses of antibiotic powders. J Biomed Mater Res 1976;10:929–938. 993228 10.1002/jbm.820100610

[16] Postak PD , Greenwald AS. The influence of antibiotics on the fatigue life of acrylic bone cement. J Bone Joint Surg (Am) 2006;88:148–155. 10.2106/JBJS.F.00586 17142444

[17] Joo H‐S , Chan JL , Cheung GYC , Otto M. Subinhibitory concentrations of protein synthesis‐inhibiting antibiotics promote increased expression of the agr virulence regulator and production of phenol‐soluble modulin cytolysins in community‐associated methicillin‐resistant *Staphylococcus aureus* . Antimicrob Agents Chemother 2010;54:4942–4944. 20713669 10.1128/AAC.00064-10 PMC2976111

[18] Zhang L , Gu FX , Chan JM , Wang AZ , Langer RS , Farokhzad OC. Nanoparticles in medicine: Therapeutic applications and developments. Clin Pharmacol Ther 2008;83:761–769. 17957183 10.1038/sj.clpt.6100400

[19] Sharma A , Sharma US. Liposomes in drug delivery: Progress and limitations. Int J Pharm 1997;154:123–140.

[20] Chang HI , Yeh MK. Clinical development of liposome‐based drugs: Formulation, characterization, and therapeutic efficacy. Int J Nanomed 2012;7:49–60. 10.2147/IJN.S26766 PMC3260950 22275822

[21] Gregoriadis G , Florence AT. Liposomes in drug delivery. Clinical, diagnostic and ophthalmic potential. Drugs 1993;45:15–28. 10.2165/00003495-199345010-00003 7680982

[22] Hsieh P , Tai C , Lee P , Chang Y. Liquid gentamicin and vancomycin in bone cement: A potentially more cost‐effective regimen. J Arthroplast 2009;24:125–130. 10.1016/j.arth.2008.01.131 18534439

[23] Seldes RM , Winiarsky R , Jordan LC , Baldini T , Brause B , Zodda F , Sculco TP . Liquid gentamicin in bone cement: A laboratory study of a potentially more cost‐effective cement spacer. J Bone Joint Surg (Am) 2005;87:268–272. 15687146 10.2106/JBJS.C.00728

[24] Miller RB , McLaren AC , Leon CM , Vernon BL , McLemore R. Surfactant‐stabilized emulsion increases gentamicin elution from bone cement. Clin Orthopaed Relat Res 2010;469:2995–3001. 10.1007/s11999-011-1934-7 PMC3183202 21656316

[25] Nishio Ayre WN , Birchall JC , Evans SL , Denyer SP ; Intellectual Property Office, Newport. Liposomal Drug Delivery System for Bone Cements. United Kingdom, PCT/GB2014/052085. 9th July 2014 Available at: http://patentscope.wipo.int/search/en/detail.jsf?docId=WO2015004450. [accessed 10th March 2015]

[26] Institution BS . ISO5833:2002 Implants for surgery: Acrylic resin cements.2002 Available at: http://www.iso.org/iso/catalogue_detail.htm?csnumber=30980 [accessed 10th March 2015]

[27] Davis EJR , International A . Handbook of Materials for Medical Devices. ASM International Materials Park, OH; 2003.

[28] Sampath SS , Robinson DH. Comparison of new and existing spectrophotometric methods for the analysis of tobramycin and other aminoglycosides. J Pharm Sci 1990;79:428–431. 2352163 10.1002/jps.2600790514

[29] Zhang X , Wyss UP , Pichora D , Goosen MFA. Biodegradable controlled antibiotic release devices for osteomyelitis: Optimization of release properties. J Pharm Pharmacol 1994;46:718–724. 7837040 10.1111/j.2042-7158.1994.tb03890.x

[30] Korsmeyer RW , Gurny R , Doelker E , Buri P , Peppas NA. Mechanisms of solute release from porous hydrophilic polymers. Int J Pharm 1983;15:25–35. 10.1002/jps.2600721021 6644570

[31] Higuchi T. Rate of release of medicaments from ointment bases containing drugs in suspension. J Pharm Sci 1961;50:874–875. 13907269 10.1002/jps.2600501018

[32] Noyes AA , Whitney WR. The rate of solution of solid substances in their own solutions. J Am Chem Soc 1897;19:930–934.

[33] Lindner WD , Lippold BC. Drug release from hydrocolloid embeddings with high or low susceptibility to hydrodynamic stress. Pharm Res 1995;12:1781–1785. 8592686 10.1023/a:1016238427313

[34] Evans S. Fatigue crack propagation under variable amplitude loading in PMMA and bone cement. J Mater Sci Mater Med 2007;18:1711–1717. 17483908 10.1007/s10856-007-3021-x

[35] Institution BS. ISO13586:2000 Plastics: Determination of fracture toughness (Gic and Kic). Linear elastic fracture mechanics (LEFM) approach; 2000 Available at: http://www.iso.org/iso/catalogue_detail.htm?csnumber=22321 [accessed 10th March 2015]

[36] Institution BS. ISO6507:2005 Metallic materials—Vickers hardness test—Part 1: Test method; 2005 Available at: http://www.iso.org/iso/catalogue_detail.htm?csnumber=37746 [accessed 10th March 2015]

[37] International ASfTaM. ASTM E399 ‐ 09e2 Standard Test Method for Linear‐Elastic Plane‐Strain Fracture Toughness of Metallic Materials. ASTM International; 2009 Available at: http://www.astm.org/Standards/E399.htm [accessed 10th March 2015]

[38] van de Belt H , Neut D , Schenk W , van Horn JR , van der Mei HC , Busscher HJ. Gentamicin release from polymethylmethacrylate bone cements and *Staphylococcus aureus* biofilm formation. Acta Orthop Scand 2000;71:625–629. 11145392 10.1080/000164700317362280

[39] Poelstra KA , Busscher HJ , Schenk W , van Horn JR , van der Mei HC. Effect of gentamicin loaded PMMA bone cement on *Staphylococcus aureus* biofilm formation. Biofouling 1999;14:249–254.

[40] Ayre WN , Denyer SP , Evans SL. Ageing and moisture uptake in polymethyl methacrylate (PMMA) bone cements. J Mech Behav Biomed Mater 2014;32:76–88. 24445003 10.1016/j.jmbbm.2013.12.010 PMC3988952

[41] Cunningham B , McLaren AC , Pauken C , McLemore R. Liposomal formulation increases local delivery of amphotericin from bone cement: A pilot study. Clin Orthop Relat Res 2012;470:2671–2676. 22467417 10.1007/s11999-012-2317-4 PMC3442012

[42] Mugabe C , Azghani AO , Omri A. Liposome‐mediated gentamicin delivery: Development and activity against resistant strains of *Pseudomonas aeruginosa* isolated from cystic fibrosis patients. J Antimicrob Chemother 2005;55:269–271. 15590716 10.1093/jac/dkh518

[43] MacLeod DL , Prescott JF. The use of liposomally entrapped gentamicin in the treatment of bovine *Staphylococcus aureus* mastitis. Can J Vet Res 1988;52:445–450. 3196975 PMC1255489

[44] Kuhn AT , Wilson AD. The dissolution mechanisms of silicate and glass‐ionomer dental cements. Biomaterials 1985;6:378–382. 3910125 10.1016/0142-9612(85)90096-1

[45] Mugabe C , Halwani M , Azghani AO , Lafrenie RM , Omri A. Mechanism of enhanced activity of liposome‐entrapped aminoglycosides against resistant strains of *Pseudomonas aeruginosa* . Antimicrob Agents Chemother 2006;50:2016–2022. 16723560 10.1128/AAC.01547-05 PMC1479138

[46] Hoey D , Taylor D. The effect of mixing technique on fatigue of bone cement when stress concentrations are present. Int J Nano Biomater 2010;3:36–48.

[47] Evans S. Effects of porosity on the fatigue performance of polymethyl methacrylate bone cement: An analytical investigation. Proc Inst Mech Eng H 2005;220:1–10. 10.1243/095441105X69024 16459441

[48] Hoey D , Taylor D. Statistical distribution of the fatigue strength of porous bone cement. Biomaterials 2009;30:6309–6317. 19699519 10.1016/j.biomaterials.2009.07.053

[49] Cho K , JaeHo Y , Park CE. The effect of interfacial adhesion on toughening behaviour of rubber modified poly(methyl methacrylate). Polymer 1997;38:5161–5167.

[50] Puckett AD , Roberts B , Bu L , Mays JW. Improved orthopaedic bone cement formulations based on rubber toughening. Crit Rev Biomed Eng 2000;28:457–461. 11108215 10.1615/critrevbiomedeng.v28.i34.180

[51] Lalande L , Plummer CJG , Manson JE , Gerard P. Microdeformation mechanisms in rubber toughened PMMA and PMMA‐based copolymers. Eng Fract Mech 2006;73:2413–2426.

[52] Lazzeri A , Bucknall CB. Dilatational bands in rubber‐toughened polymers. J Mater Sci 1993;28:6799–6808.

[53] Todo M , Takahashi K , Ben Jar P , Beguelin P. Toughening mechanisms of rubber toughened PMMA. JSME Int J Ser A 1999;42:585–591.

[54] Hwang JF , Manson JA , Hertzberg RW , Miller GA , Sperling LH. Fatigue crack propagation of rubber‐toughened epoxies. Polym Eng Sci 1989;29:1477–1487.

